# Clinically guided adaptive contrast adjustment for fetal plane classification: a modular plug-and-play solution

**DOI:** 10.3389/fphys.2025.1689936

**Published:** 2025-11-13

**Authors:** Yang Chen, Sanglin Zhao, Baoyu Chen, Måns Gustaf

**Affiliations:** 1 School of Mathematics and Statistics, Xiamen University of Technology, Xiamen, China; 2 Hunan University of Finance and Economics, Changsha, China; 3 Dongguan University of Technology, Dongguan, China; 4 School of Business, Society and Engineering, Mälardalens University, Västerås, Sweden

**Keywords:** fetal ultrasound, clinically-inspired module, adaptive contrast adjustment, robust medical image analysis, plug and play (PnP)

## Abstract

Fetal ultrasound standard plane recognition plays a vital role in ensuring accurate prenatal assessment but remains challenging due to intrinsic factors such as poor tissue contrast, indistinct anatomical boundaries, and variability in image quality caused by operator differences. To address these issues, we introduce a plug-and-play Adaptive Contrast Adjustment Module (ACAM), inspired by how clinicians manually adjust image contrast to highlight clearer structural cues. The proposed module integrates a lightweight, texture-aware subnetwork that learns to generate clinically meaningful contrast parameters, producing multiple contrast-enhanced representations of the same image through a differentiable transformation process. These enhanced views are then fused within subsequent classifiers to enrich discriminative features. Experiments conducted on a multi-center dataset containing 12,400 fetal ultrasound images across six anatomical planes demonstrate consistent performance gains: the accuracy of lightweight models rises by 2.02%, conventional architectures by 1.29%, and state-of-the-art models by 1.15%. The key novelty of ACAM lies in its content-adaptive and clinically aligned contrast modulation, which replaces random preprocessing with physics-guided transformations mimicking sonographers’ diagnostic workflows. By leveraging multi-view contrast fusion, our approach enhances robustness against image quality variations and effectively links low-level texture cues with high-level semantic understanding, offering a new framework for medical image analysis in realistic clinical settings. Our code is available at: https://github.com/sysll/ACAM.

## 1 Introduction

Ultrasound offers several advantages, including safety, convenience, non-invasiveness, and the absence of ionizing radiation, which has led to its widespread application in critical areas such as prenatal fetal screening Maher and Seed (2024); Wittek et al. (2025); Al-Dahim et al. (2024); Miller et al. (2020); Wang (2018). The acquisition of standardized fetal ultrasound planes is essential for improving diagnostic precision and minimizing the risk of overlooking severe fetal abnormalities. Nevertheless, achieving this standardization remains challenging: it requires operators to have comprehensive knowledge of fetal anatomy, while clinical expertise and equipment conditions may sometimes be inadequate. Furthermore, the increasing complexity of screening settings, the rising demand for fetal examinations, and the shortage of skilled ultrasound practitioners make manual acquisition of high-quality planes even more difficult. In this context, there is a pressing need for automated recognition systems to support sonographers in efficiently and accurately identifying standard fetal trunk planes. These systems can help reduce missed diagnoses, improve workflow efficiency, and provide more reliable and safer technical assistance for prenatal evaluation.

Deep learning has demonstrated remarkable capabilities and has been widely applied across various domains Cai et al. (2024); Zhu et al. (2024); Ou et al. (2024); Mykula et al. (2024); Zhao et al. (2024a); Zhao et al. (2024b); Jin et al. (2025); Zhao et al. (2025). In recent years, there has been growing interest in algorithms for fetal ultrasound plane analysis Zhu et al. (2025b), Zhu et al. (2025a); Boumeridja et al. (2025); Montero et al. (2021); Yousefpour Shahrivar et al. (2023); Krishna and Kokil (2024); Krishna and Kokil (2023); Fiorentino et al. (2025a); Migliorelli et al. (2024). However, most studies primarily focus on feature extraction modules, emphasizing information in intermediate network layers or increasing dataset size to improve model performance. For example, Zhu et al. (2025b) aimed to optimize pooling layer performance; while insightful, this approach overlooks the impact of the input layer. Similarly, Montero et al. (2021) employed generative adversarial networks (GANs) to generate additional training images, thereby enlarging the dataset. Only a few studies consider the interaction between the model and the input image in relation to image quality. For instance, Zhu et al. (2025a) highlighted the importance of selecting appropriate contrast and gain for medical image performance and proposed an attention mechanism to focus on regions with critical gain. However, in their approach, contrast and gain are fixed rather than adaptively generated, which limits the model’s capability. To address these limitations, we propose an Adaptive Contrast Adjustment Module (ACAM) that dynamically adjusts image contrast based on image content. By generating multiple contrast-enhanced versions and fusing their information, the module not only enriches texture representations but also significantly improves the classification accuracy of complex fetal plane images.

Our approach is motivated by the practical workflow of clinicians when identifying fetal planes during ultrasound examinations. In routine practice, sonographers often manipulate image contrast to emphasize key anatomical structures, which helps produce clearer and more discriminative images Smith and Lopez (1982); Mehta et al. (2017). Drawing from this idea, we incorporate an adaptive contrast adjustment module into our model. Specifically, the input image is first processed by a decision network that predicts K potential contrast parameters. These predicted parameters are then mapped to a predefined fixed range using a differentiable function to ensure numerical stability and preserve trainability. Using these contrast parameters, the input image is transformed to produce K contrast-enhanced variants, effectively introducing multiple perspectives or styles during training. These enhanced images are then passed through a convolutional neural network for feature extraction and classification. Since contrast adjustment relies more on local texture information rather than high-level semantic cues, we employ a shallow convolutional network as the decision module. This network captures fine-grained details, such as edges and textures, to generate the contrast parameters. This design provides both interpretability and generalization benefits. The parameters directly control image brightness and contrast, making the transformations intuitive and visually interpretable, in contrast to black-box manipulations of abstract features. Additionally, by explicitly generating multiple contrast scenarios, the model learns representations that are more robust to variations in illumination and contrast, which improves generalization across different acquisition settings or imaging domains.

Furthermore, our module adopts a plug-and-play architecture and is applied solely to the lower layers of the network, allowing for easy integration. We evaluated its effectiveness by embedding it into conventional robust models, lightweight networks, and cutting-edge architectures, performing ablation studies to quantify its impact. Comparative experiments were subsequently conducted against eight baseline models. The results indicate that incorporating our module consistently improves performance. The main benefits of the module are summarized as follows:

- The module emulates the way clinicians adjust image contrast, allowing adaptive generation of multiple images with different contrast levels. This enables the model to learn from diverse representations, enhancing its sensitivity to fine details and improving overall robustness.
- In our framework, a shallow convolutional network first extracts local texture information from the input image. Using these features, the network predicts several candidate contrast values, which are then applied to enhance the image and enrich feature representation.
- We incorporated the module into lightweight CNNs, conventional robust models, and state-of-the-art architectures, performing comprehensive evaluations. Comparative experiments, ablation studies, and heatmap visualizations confirm that the module consistently boosts model performance and generalizability.

## 2 Methods

### 2.1 Linear contrast

Image contrast enhancement can be achieved through either linear or nonlinear gray-level transformations, with the basic goal of stretching or compressing the distribution range of pixel intensities, thereby emphasizing the intensity differences across regions of the image. Let the original grayscale image be denoted as I(x,y) , where (x,y) represents the pixel location in the image. A commonly used linear contrast adjustment method can be formulated as shown in Equation 1:

$$I^{\prime }\left(x,y\right)=\alpha \cdot \left(I\left(x,y\right)-\mu \right)+\mu ,$$ (1)

where $I^{\prime }\left(x,y\right)$ represents the adjusted pixel intensity, α>0 is the contrast scaling factor (typically referred to as the contrast gain), and μ is the mean intensity (brightness center) of the image, defined as shown in Equation 2:

$$\mu =\frac{1}{HW}\overset{H}{\underset{x=1}{\sum }}\overset{W}{\underset{y=1}{\sum }}I\left(x,y\right),$$ (2)

with H and W denoting the image height and width, respectively.

When α>1 , the contrast of the image is increased, whereas α<1 reduces the contrast.

### 2.2 The mechanism of the ACAM module

**FIGURE 1:**
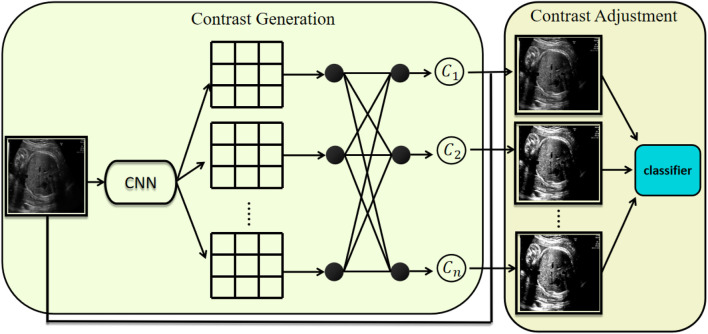
Architecture of the proposed module. It consists of two components: contrast generation and contrast adjustment. The contrast generation component predicts k distinct contrast parameters, which are subsequently used by the contrast adjustment component to transform the input image accordingly.

The structure of our module is illustrated in Figure 1. First, the input is a grayscale image with dimensions [1, H, W]. The first step of the model is to generate a set of contrast values from this image for subsequent processing. We posit that contrast prediction primarily relies on the detailed information within the image rather than semantic-level features. Therefore, this module employs a shallow architecture composed of convolutional layers, a global average pooling layer, and fully connected layers. This design is chosen because shallow convolutional neural networks are more adept at extracting high-frequency detail information from images, whereas deeper convolutions mainly capture semantic features. Moreover, using a low-level structure introduces fewer parameters. The predicted contrast values are then mapped to the range [1, 3] to align with the adjustment range typically used by clinicians. This process can be expressed as Equation 3.

$$C=\text{Function}\left(\text{FC}\left(\text{GAP}\left(\text{Conv}\left(\text{Image}\right)\right)\right)\right)\;$$ (3)

where $C=\left[c_{1},c_{2},\ldots ,c_{k}\right]$ . The Function(x) function is shown in Equation 4.

$$\text{Function}\left(x\right)=1+2\frac{1}{1+e^{-x}}\;$$ (4)

These contrast values are then fed into the model as contrast parameters. The specific formula is given in Equation 5.

$$I_{\mathrm{kij}}=c_{k}\left(I_{\mathrm{kij}}-\frac{1}{HW}\underset{ij}{\sum }I_{\mathrm{kij}}\right)+\frac{1}{HW}\underset{ij}{\sum }I_{\mathrm{kij}}$$ (5)

Where $I_{\mathrm{kij}}$ denotes the pixel at row i , column j of the k -th image. The final output (I) is with dimensions of [K×H×W] , representing k images generated under k different contrast conditions. These images constitute the output of our module and serve as input to subsequent decision-making models such as MedMamba. Clinicians typically begin by adjusting image contrast to enhance clarity before proceeding to in-depth analysis for diagnostic classification. Our ACAM module mimics this contrast enhancement process to optimize visual clarity, operating primarily on low-level texture information. In contrast, downstream decision-making models such as MedMamba simulate the clinician’s diagnostic reasoning process, which necessitates the extraction of high-level semantic features, so they have deeper layers.

### 2.3 Implementation details

**FIGURE 2:**
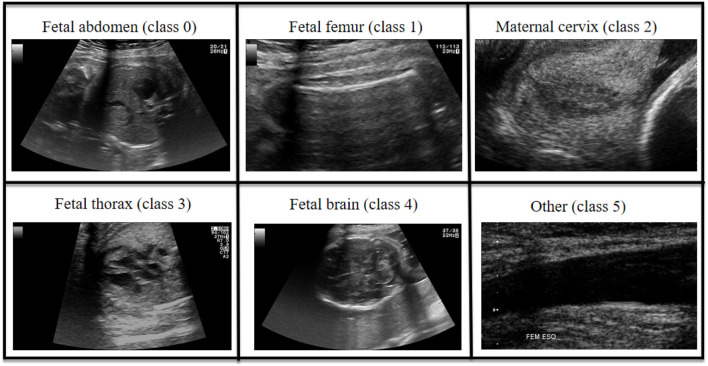
Sample images from each class of the dataset.

This study is based on a large-scale prenatal screening ultrasound image dataset Burgos-Artizzu et al. (2020), which was collected from two hospitals and encompasses multiple operators as well as different ultrasound device models. All images were manually annotated by a single obstetrics expert and categorized into six classes: four commonly used fetal standard planes (abdominal, brain, femur, and thoracic), the maternal cervix plane for preterm screening, and a general class including other less common planes. The names of these standard planes and their corresponding encoded categories are shown in Figure 2. The number of images for each standard plane category is shown in the Table 1. The final dataset comprises over 12,400 images from 1,792 patients, and it was split into training and test sets at a ratio of 7:3. All experiments were conducted using Python 3.9 and the PyTorch 2.0.1+cu117 framework, on a system equipped with an Intel i7-12650H processor and an NVIDIA RTX2080Ti GPU. Detailed settings of the model parameters and baseline models are provided in Table 2, with the number of generated contrast images n set to 10.

**TABLE 1:** The number of each fetal ultrasound standard plane in the dataset.

Plane	Number
Fetal abdomen (class 0)	711
Fetal femur (class 1)	1,040
Maternal cervix (class 2)	1,626
Fetal thorax (class 3)	1718
Fetal brain (class 4)	3,092
Other (class 5)	4,213

**TABLE 2:** Hyperparameter settings used during model training.

Hyperparameter	Value
Batch size	64
Epoch	20
Lr	0.001
Optimizer	Adam (Bock and Weiß, 2019)
Loss function	Crossentropy (Mao et al., 2023)

We justify our hyperparameter choices as follows. The contrast range is set to [1,3] , based on clinical practice. The study Zhu et al. (2025c) discussed the changes in ultrasound images with contrast ranging from 1 to 2.5, illustrating the advantages of this range. Clinicians typically adjust image contrast within this range to enhance the visibility of anatomical structures while avoiding image distortion—values below 1.0 excessively compress the dynamic range, whereas values above 3.0 may cause key regions to become oversaturated. The number of contrast variants is configured at K=10 , taking into account clinical practices. While clinicians may experiment with various contrast adjustments for a single image, they seldom employ an excessive number. In the study by Zhu et al. (2025a), nine contrast groups with fixed gain settings were used, which is close to our setting. Therefore, choosing 10 variants strikes a balance between computational efficiency and feature diversity. For the network architecture, we use a shallow convolutional network for contrast prediction to extract texture features, as contrast adjustment mainly relies on low-level image statistics rather than high-level semantic information. Studies Zhang et al. (2019); Gao et al. (2020); Cimpoi et al. (2015) have all highlighted the role of shallow neural networks in extracting texture information. In terms of training configuration, the batch size is set to 64 to efficiently utilize GPU memory and follow the powers-of-two convention; the learning rate is set to 0.001 to ensure stable convergence; and the model is trained for 20 epochs to achieve sufficient convergence while avoiding overfitting. This setup is based on the experience reported in previous studies on related datasets Burgos-Artizzu et al. (2020); Zhu et al. (2025a); Montero et al. (2021).

## 3 Results

### 3.1 Evaluation metrics and baseline models

In this study, multiple widely adopted evaluation metrics are employed to systematically analyze model performance. Accuracy (ACC) reflects the overall correctness of predictions; however, it may be misleading in scenarios with imbalanced class distributions. Recall measures the model’s ability to correctly identify positive samples, which is particularly crucial in medical image analysis, as higher recall helps reduce the risk of missed diagnoses. Precision evaluates the proportion of predicted positive samples that are truly positive, thereby reducing the likelihood of false alarms. The F1-score, defined as the harmonic mean of precision and recall, provides a balanced assessment of both metrics.

Here, we denote the standard confusion matrix terms as follows:

-
TP (True Positive): number of correctly predicted positive samples,
-
TN (True Negative): number of correctly predicted negative samples,
-
FP (False Positive): number of negative samples incorrectly predicted as positive,
-
FN (False Negative): number of positive samples incorrectly predicted as negative.

Based on these definitions, the metrics are computed as Equations 6–9:

$$\text{Accuracy}=\frac{TP+TN}{TP+TN+FP+FN}$$ (6)

$$\text{Precision}=\frac{TP}{TP+FP}$$ (7)

$$\text{Recall}=\frac{TP}{TP+FN}$$ (8)

$$F1=\frac{2\times \text{Precision}\times \text{Recall}}{\text{Precision}+\text{Recall}}$$ (9)

In addition, to comprehensively characterize the model’s classification capability across different decision thresholds, we introduce the Receiver Operating Characteristic (ROC) curve and employ the Area Under the Curve (AUC) as a performance indicator. Similarly, the Precision–Recall (PR) curve is utilized to illustrate prediction accuracy at varying recall levels, with the Average Precision (AP) computed to intuitively reflect the model’s ability in target detection tasks.

To evaluate the effectiveness of the proposed model, we compare it against several established deep learning architectures, including EfficientNet Kashyap et al. (2023), InceptionV3 Szegedy et al. (2016), VGG Gunasekaran and Vivekasaran (2024), ResNet Xu et al. (2023), MobileNet Han et al. (2022), ShuffleNet Hou et al. (2025), ConvNeXt Sangeetha and Geetha (2024), MedMamba Bansal et al. (2024), EfficientVMamba Pei et al. (2025), OrthoNets Salman et al. (2023), and Efficientvit Liu et al. (2023).

### 3.2 Comparison experiment

The performance comparison of the models is presented in Table 3. As shown, all evaluated models—ranging from lightweight networks such as ShuffleNet, MobileNet, and EfficientNet to traditional robust architectures including ResNet, VGG, InceptionV3, and ConVNeXt, as well as state-of-the-art deep learning models such as MedMamba variants and EfficientViT—achieved strong performance on the test set, with overall accuracy consistently exceeding the 90% baseline. Specifically, classical architectures like EfficientNet and InceptionV3 achieved top-1 accuracies of 92.26% and 92.32%, respectively, while MobileNet and VGG attained slightly lower accuracies of 90.27% and 90.73%. In addition to these baseline models, we evaluated RCJ-based models, which use Random Contrast Jittering as a data augmentation strategy. The incorporation of RCJ generally led to modest improvements across different backbones. For instance, RCJ-ResNet improved the accuracy from 91.72% to 92.02%, RCJ-MedMamba increased from 92.32% to 92.48%, and RCJ-ShuffleNet improved from 89.28% to 89.39%. These results indicate that contrast-based augmentation contributes to better robustness against intensity variations. The proposed ACAM module (Adaptive Contrast Adjustment Module), when integrated into different backbone networks, consistently improved model performance. ACAM-MedMamba achieved the highest accuracy of 93.47% and an F1-score of 93.47%, surpassing both the original MedMamba (92.32% accuracy, 92.36% F1-score) and RCJ-MedMamba (92.48% accuracy, 92.53% F1-score). Similarly, ACAM-ResNet improved accuracy from 91.72% to 93.01%, and ACAM-ShuffleNet increased accuracy from 89.28% to 91.30%. These results demonstrate the generalization capability of the ACAM module across different architectures. Overall, Table 3 shows that ACAM not only outperforms baseline and RCJ-enhanced models but also effectively enhances feature discrimination and complements existing data augmentation strategies, providing a robust approach for medical image classification tasks.

**TABLE 3:** Ablation study results of our module integrated into different models, as well as comparisons with other models.

Model	ACC	Precision	Recall	F1-score
ACAM-ResNet	0.9301	0.9318	0.9301	0.9300
RCJ-ResNet	0.9202	0.9216	0.9202	0.9183
ResNet	0.9172	0.9203	0.9172	0.9167
ACAM-MedMamba	**0.9347**	**0.9351**	**0.9347**	**0.9347**
RCJ-Medmamba	0.9248	0.9284	0.9248	0.9253
MedMamba	0.9232	0.9266	0.9232	0.9236
ACAM-ShuffleNet	0.9130	0.9125	0.9130	0.9125
RCJ-ShuffleNet	0.8939	0.8932	0.8939	0.8908
ShuffleNet	0.8928	0.8920	0.8928	0.8894
EfficientNet	0.9226	0.9237	0.9226	0.9226
InceptionV3	0.9232	0.9240	0.9232	0.9216
MobileNet	0.9027	0.9076	0.9027	0.9036
VGG	0.9073	0.9100	0.9073	0.9075
ConVNeXt	0.8923	0.8951	0.8923	0.8899
EfficientVMamba	0.8953	0.8943	0.8953	0.8921
OrthoNets	0.9218	0.9253	0.9218	0.9223
Efficientvit	0.9205	0.9238	0.9205	0.9209

The best-performing values are highlighted in bold.

### 3.3 Ablation study

The results of the ablation study are summarized in Table 3. It can be seen that, regardless of whether the backbone is a traditional model (ResNet), a lightweight model (ShuffleNet), or a state-of-the-art model (MedMamba), integrating the proposed module leads to a significant performance improvement, with an average gain of 1.48%. This consistent enhancement across different architectures demonstrates the effectiveness and generality of the proposed module.

**FIGURE 3:**
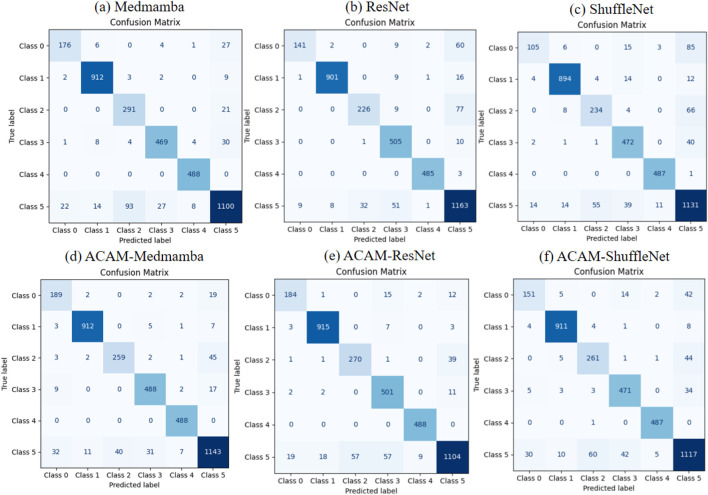
Comparison of confusion matrices for three models before and after integrating the proposed module. **(a–c)** show the classification performance of the baseline models, while **(d–f)** illustrate the improvements achieved after incorporating the module.

A comparison of confusion matrices, as shown in Figure 3, reveals that the ACAM module consistently improves classification performance across lightweight models (ShuffleNet), traditional models (ResNet), and state-of-the-art models (MedMamba). In particular, the classification accuracy for classes 0 and 1 is significantly enhanced in all models, with a substantial reduction in misclassifications. For class 5, most cases also show improved precision after module integration. These results highlight that ACAM can robustly optimize feature discrimination for both common and challenging classes across various backbone networks. Furthermore, the module effectively mitigates inter-class confusion, especially in models prone to overfitting or with limited representational capacity, confirming its generalization and robustness.

**FIGURE 4:**
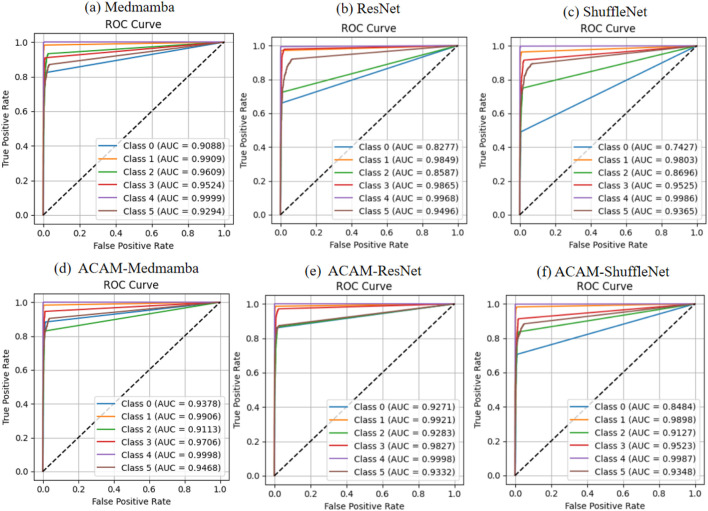
Comparison of ROC curves for three models before and after integrating the proposed module. **(a–c)** depict the classification performance of the baseline models, while **(d–f)** demonstrate the improvements achieved after incorporating the module.

**FIGURE 5:**
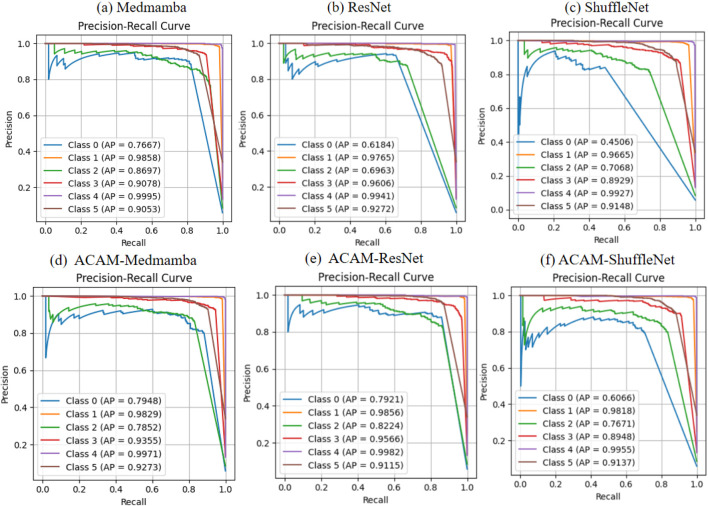
Comparison of PR curves for three models before and after integrating the proposed module. **(a–c)** Illustrate the classification performance of the baseline models, while **(d–f)** demonstrate the improvements obtained after incorporating the module.

As shown in Figure 4, the ROC curve analysis demonstrates that integrating the ACAM module significantly improves the classification performance of various models. Across the lightweight ShuffleNet, the conventional ResNet, and the advanced MedMamba, the trade-off between true positive rate (TPR) and false positive rate (FPR) is markedly enhanced for most classes. Specifically, after incorporating ACAM, MedMamba achieves notable AUC improvements of approximately 3%, 2%, and 2% for classes 0, 3, and 5, respectively; ResNet shows clear AUC gains of about 10%, 7%, and 1% for classes 0, 2, and 3; while ShuffleNet also exhibits appreciable AUC improvements of around 10% and 4% for classes 0 and 2. These observations further validate that ACAM provides consistent AUC enhancement and robustness across different model architectures. The precision–recall (PR) curves, shown in Figure 5, indicate that the module significantly enhances classification performance for key classes. In ShuffleNet, ACAM effectively improves the balance between precision and recall for classes 0, 1, and 2, with AP increases of approximately 15%, 2%, and 6%, respectively. For ResNet, notable improvements are observed in classes 0, 1, and 2, with AP increases of about 18%, 2%, and 12%. In MedMamba, classes 0, 3, and 5 clearly benefit from the module, with AP increases of roughly 3%, 3%, and 2%. These results suggest that ACAM can adaptively enhance the recognition of challenging samples according to the characteristics of different backbone networks, achieving higher recall while maintaining high precision, thereby demonstrating its broad applicability and effectiveness in improving classification performance.

### 3.4 Heatmap-based and t-SNE visualization and analysis of detailed classification results

**FIGURE 6:**
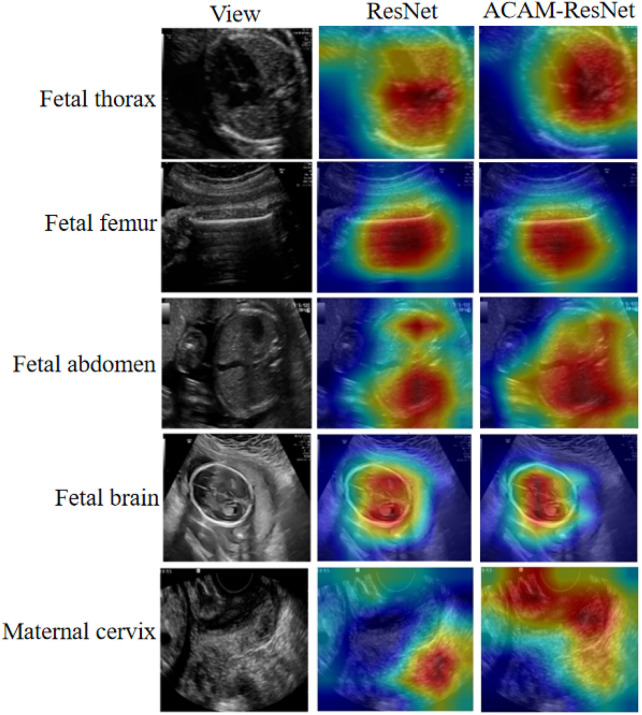
Heatmap visualizations of the ResNet model before and after integrating our module, illustrated on five representative image categories.

To further assess the effectiveness of the proposed ACAM module, we utilized the Grad-CAM technique to visualize the model’s attention regions. It should be noted that the visualizations are primarily based on ResNet, because Grad-CAM depends on the spatial feature maps of convolutional layers, which allow the generation of heatmaps with improved spatial alignment and interpretability in convolutional networks. As illustrated in Figure 6, the first column displays the original ultrasound images, while the second and third columns show the heatmaps produced by the baseline ResNet and the ACAM-enhanced ResNet (ACAM-ResNet), respectively. The results suggest that, unlike the baseline ResNet where attention areas are often scattered or misaligned with the relevant anatomical structures, ACAM-ResNet can concentrate more precisely on clinically important regions. For fetal thoracic planes, the baseline ResNet tends to distribute attention broadly across the thoracic cavity, whereas ACAM-ResNet significantly improves focus on critical organs, such as the heart and lungs. In the fetal femur planes, the baseline model may assign attention to surrounding soft tissues, but the ACAM-enhanced network accurately highlights the femoral shaft. In abdominal plane analysis, ACAM-ResNet shows more distinct attention toward structures such as the stomach bubble and umbilical cord insertion point, whereas the heatmaps from the baseline model are often diffuse. For fetal brain planes, the enhanced model clearly targets the lateral ventricles and midline structures, avoiding distraction from irrelevant brain regions. Moreover, in maternal cervical planes, ACAM-ResNet effectively emphasizes the internal cervical os and the cervical lumen, while the baseline model is easily diverted by adjacent tissues.

**FIGURE 7:**
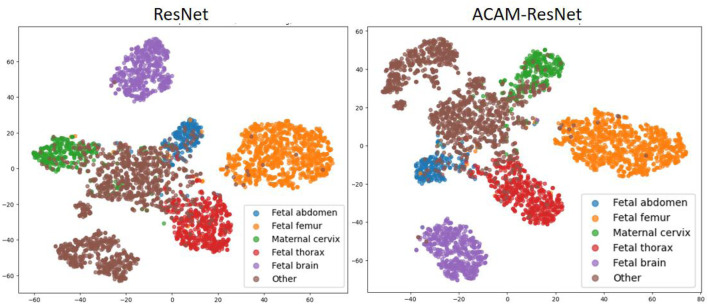
t-SNE visualization of feature embeddings extracted by ResNet and ACAM-ResNet models.

To analyze the feature distribution and inter-class relationships learned by different models, we plotted the t-SNE visualizations as shown in Figure 7. Analysis of the t-SNE visualization reveals that the feature clusters corresponding to the fetal brain and femur categories exhibit the most distinct separation, demonstrating clear isolation from other categories in the embedded space. With the exception of the “Other” category, all remaining classes maintain reasonably well-defined spatial boundaries. In the baseline ResNet model prior to integrating our ACAM module, feature representations of different categories appear in closer proximity, with substantial overlap observed particularly between the fetal thorax and “Other” categories. Following the incorporation of the ACAM module, the feature distributions show noticeable improvement in category separation, as evidenced by the more dispersed spatial arrangement of clusters. This observed expansion in inter-class distances demonstrates the module’s effectiveness in enhancing feature discriminability.

As shown in Table 4, the model performs well on most standard fetal planes, with the highest recognition achieved for the fetal femur (F1 = 0.9764) and fetal brain (F1 = 0.9869). This can be attributed to the distinctive anatomical features of these regions—specifically, the femur’s linear hyperechoic structure and the brain’s midline pattern—which provide stable cues for the model’s discrimination. However, the recall for the maternal cervix plane is relatively low (0.7442), primarily due to the following factors: first, the cervix exhibits significant morphological variation across different gestational weeks, ranging from a cylindrical to a funnel shape, resulting in large intra-class differences; second, even slight deviations in the probe angle can lead to incomplete visualization of the endometrial line, causing some positive samples to lack critical discriminative features; additionally, acoustic artifacts from the cervix plane overlapping with parts of the vaginal fornix introduce feature confusion. The precision for the fetal abdomen plane is also relatively low (0.8224), mainly because the abdominal plane often contains multiple solid organs (e.g., liver, intestines) with mixed echogenic patterns, which vary considerably across gestational ages and fetal positions. In particular, when the fetal abdomen includes amniotic fluid regions, it can be acoustically confused with fluid-filled structures in the thoracic cavity. Despite these challenges, the model maintains stable performance on most standard planes, demonstrating its ability to handle the inherent variability in fetal ultrasound images. Future work will incorporate attention mechanisms and domain adaptation strategies to further enhance the model’s discriminative capability on difficult samples.

**TABLE:** The detailed performance of our integrated model on each fetal plane category based on MedMamba.

Class	Recall	Precision	F1-score
Fetal abdomen	0.8756	0.8224	0.8482
Fetal femur	0.9702	0.9828	0.9764
Maternal cervix	0.7442	0.9327	0.8279
Fetal thorax	0.9343	0.9089	0.9214
Fetal brain	0.9741	1.0000	0.9869
Other	0.9267	0.8703	0.8976

## 4 Discussion

### 4.1 Module significance and comparison with existing methods

In fetal ultrasound standard plane recognition, most methods rely on CNNs for texture and edge feature extraction Venkatareddy et al. (2024); Diniz et al. (2020); Wang et al. (2021), assuming input images of stable quality and moderate contrast. In clinical practice, however, factors such as fetal position, gestational age, device settings, and operator habits often cause substantial contrast variations, obscuring critical anatomical details. Clinicians typically adjust contrast to highlight essential structures, inspiring the design of our ACAM. Unlike conventional data augmentation, which applies random transformations without adapting to image content, ACAM dynamically models contrast in a content-aware manner, enhancing texture details and exploring multiple contrast perspectives. This approach preserves discriminative capability even with blurred structures or low signal-to-noise ratios. Beyond technical improvement for plane classification, ACAM reflects a paradigm aligning deep learning with clinical imaging practices, offering insights into medical AI by modeling contrast—a low-level yet clinically significant attribute.

Krishna and Kokil (2024) employed a stacked ensemble approach using three pre-trained deep CNNs: AlexNet, VGG-19, and DarkNet-19. Predictions from these networks were obtained via Softmax and random forest classifiers. In Krishna and Kokil (2023), AlexNet and VGG-19 were used to extract deep features, with a global average pooling layer as the final pooling layer for feature integration. Fusing deep features extracted from different convolutional networks enhances the overall feature representation. In contrast to their studies, which primarily focus on the diversity of extracted features, our work emphasizes adaptive adjustment of image contrast to improve image quality. Moreover, Venkatareddy et al. (2024) introduced explainable AI (XAI) methods—specifically Local Interpretable Model-agnostic Explanations (LIME)—to increase the transparency and reliability of model decisions. Our approach, however, introduces adaptive contrast generation, which not only enhances model performance but also improves the interpretability of the model design.

### 4.2 Secondary training strategy

**FIGURE 8:**
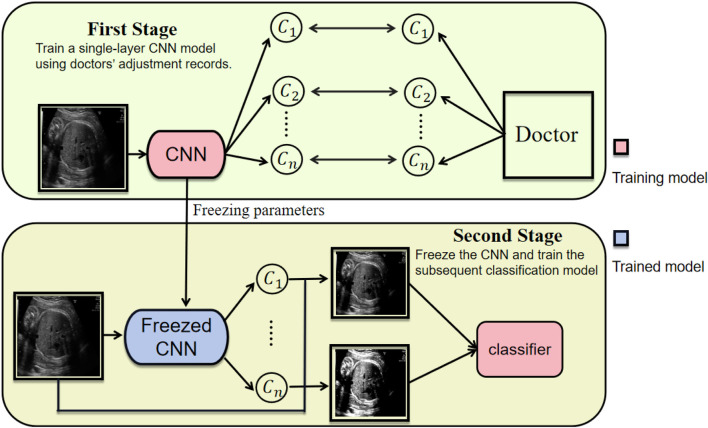
Two-stage training strategy of ACAM, contrast generation (Stage 1) and classification (Stage 2).

Our model further supports an extended application. Specifically, the system can record clinicians’ contrast adjustment operations across various fetal ultrasound planes and use these records to supervise the training of the convolutional module in the contrast generation stage (Stage 1 in Figure 8). In the subsequent classification stage, the parameters of the first convolutional layer are frozen (Stage 2 in Figure 8). The core design of ACAM intrinsically simulates the clinical decision-making process: clinicians first adjust image contrast until the plane becomes sufficiently clear, and only then proceed with diagnosis. Our two-stage strategy closely aligns with this workflow by decomposing the task into two sequential objectives—first training the model to predict contrast, and then training the classification model using the contrast-enhanced images. This staged training paradigm not only improves model performance but also enhances interpretability, as the feature generation process explicitly reflects clinicians’ operational preferences. Furthermore, the method demonstrates strong extensibility, allowing adaptation to data acquired from different devices or operators, thereby further improving robustness and clinical applicability.

### 4.3 Limitations and future directions

Although our method can automatically generate multiple contrast values from input images—enhancing the model’s sensitivity to fine-grained details—the number of generated contrast values is currently fixed. This design may limit adaptability when the model encounters extreme or previously unseen contrast variations. Future work could explore more flexible contrast generation mechanisms, such as variable-size or continuously parameterized approaches, to better capture a wider spectrum of contrast distributions and further improve robustness and generalization. Incorporating clinician adjustment records or prior clinical knowledge also represents a promising direction to enhance interpretability and clinical relevance.

Moreover, while our study demonstrates the effectiveness of ACAM on a widely used public fetal ultrasound benchmark, we acknowledge that relying on a single dataset may restrict generalizability. As highlighted by Fiorentino et al. (2025b), this dataset contains several biases, including class imbalance, demographic underrepresentation, and acquisition heterogeneity. These factors can affect model performance and may not fully reflect clinical variability in broader populations. By explicitly addressing these challenges, our work underscores the value of modules like ACAM in improving model robustness to image-level variations. Future studies will aim to validate ACAM on more diverse clinical datasets to further assess its generalizability and practical applicability in real-world settings.

## 5 Conclusion

This work presents ACAM, a novel paradigm for fetal ultrasound plane classification that fundamentally mitigates performance degradation caused by low-contrast tissue boundaries. Inspired by clinical practice, where sonographers routinely adjust image contrast to obtain clearer and more discriminative views, we incorporate this insight into the design of ACAM. By integrating contrast adjustment directly into feature learning through a dynamically parameterized module, ACAM generates anatomically meaningful multi-contrast views guided by local texture cues, significantly enhancing detail discriminability without compromising semantic extraction. Its seamless integration across convolutional, lightweight, and modern architectures demonstrates universal effectiveness, with an average accuracy gain of 1.48% validated on multi-center clinical data. Furthermore, we validated through Grad-CAM heatmaps that the proposed module enables the model to focus more on detailed information. Future work will explore physician-guided training via adjustment records and dynamic parameterization for broader contrast scenarios. ACAM provides a practical way of embedding imaging physics into deep learning pipelines, contributing to more reliable medical image analysis under heterogeneous clinical conditions.

## Data Availability

The original contributions presented in the study are included in the article/Supplementary Material, further inquiries can be directed to the corresponding author.
