# Supplementary material for: Clinically guided adaptive contrast adjustment for fetal plane classification: a modular plug-and-play solution
Source: Front Physiol. 2025 Nov 13;16:1689936. doi: 10.3389/fphys.2025.1689936 (PMC12657189; doi:10.3389/fphys.2025.1689936)
Supplement: Supplementary file 1 [file DataSheet1.zip › ACAM-main/PR1.pdf]

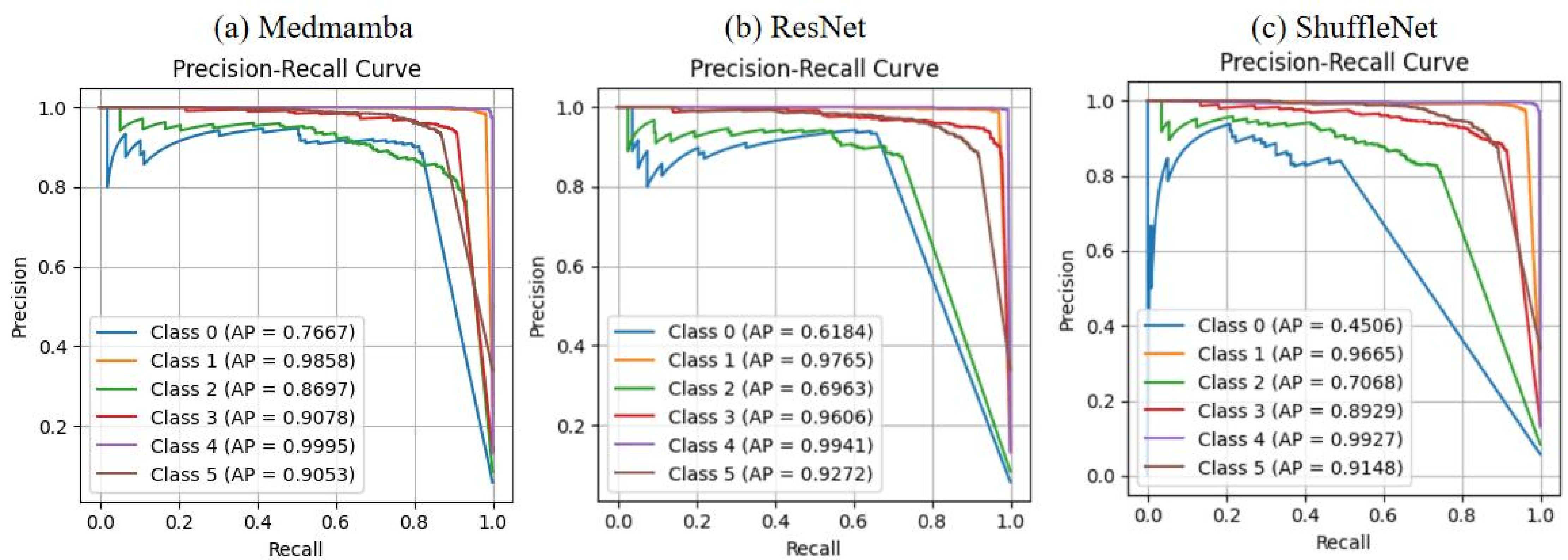
(a) Medmamba
Precision-Recall Curve
Precision
Recall
1.0
0.8
0.6
0.4
0.2
0.0
0.2
0.4
0.6
0.8
1.0
Class 0 (AP = 0.7667)
Class 1 (AP = 0.9858)
Class 2 (AP = 0.8697)
Class 3 (AP = 0.9078)
Class 4 (AP = 0.9995)
Class 5 (AP = 0.9053)
(b) ResNet
Precision-Recall Curve
Precision
Recall
Class 0 (AP = 0.6184)
Class 1 (AP = 0.9765)
Class 2 (AP = 0.6963)
Class 3 (AP = 0.9606)
Class 4 (AP = 0.9941)
Class 5 (AP = 0.9272)
(c) ShuffleNet
Precision-Recall Curve
Precision
Recall
Class 0 (AP = 0.4506)
Class 1 (AP = 0.9665)
Class 2 (AP = 0.7068)
Class 3 (AP = 0.8929)
Class 4 (AP = 0.9927)
Class 5 (AP = 0.9148)
