# Supplementary material for: Clinically guided adaptive contrast adjustment for fetal plane classification: a modular plug-and-play solution
Source: Front Physiol. 2025 Nov 13;16:1689936. doi: 10.3389/fphys.2025.1689936 (PMC12657189; doi:10.3389/fphys.2025.1689936)
Supplement: Supplementary file 1 [file DataSheet1.zip › ACAM-main/PR2.pdf]

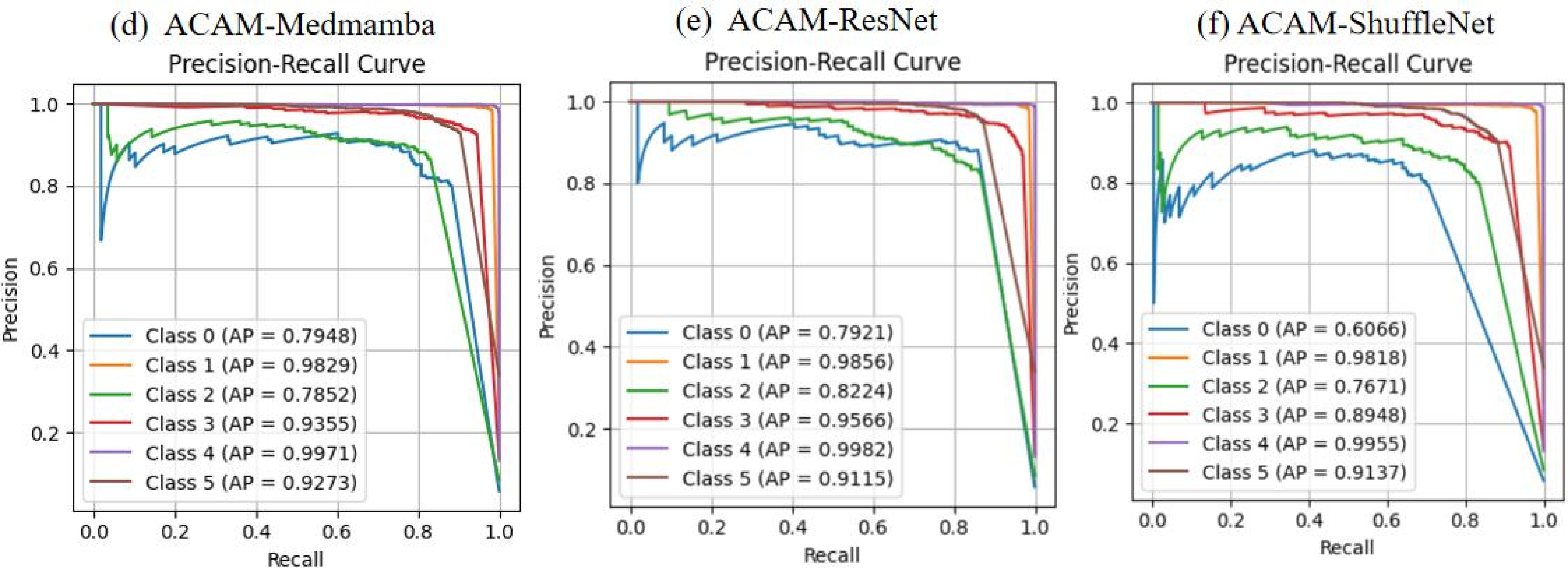
(d) ACAM-Medmamba
Precision-Recall Curve
Precision
Recall
0.0
0.2
0.4
0.6
0.8
1.0
Class 0 (AP = 0.7948)
Class 1 (AP = 0.9829)
Class 2 (AP = 0.7852)
Class 3 (AP = 0.9355)
Class 4 (AP = 0.9971)
Class 5 (AP = 0.9273)
(e) ACAM-ResNet
Precision-Recall Curve
Precision
Recall
Class 0 (AP = 0.7921)
Class 1 (AP = 0.9856)
Class 2 (AP = 0.8224)
Class 3 (AP = 0.9566)
Class 4 (AP = 0.9982)
Class 5 (AP = 0.9115)
(f) ACAM-ShuffleNet
Precision-Recall Curve
Precision
Recall
Class 0 (AP = 0.6066)
Class 1 (AP = 0.9818)
Class 2 (AP = 0.7671)
Class 3 (AP = 0.8948)
Class 4 (AP = 0.9955)
Class 5 (AP = 0.9137)
