# Supplementary material for: Clinically guided adaptive contrast adjustment for fetal plane classification: a modular plug-and-play solution
Source: Front Physiol. 2025 Nov 13;16:1689936. doi: 10.3389/fphys.2025.1689936 (PMC12657189; doi:10.3389/fphys.2025.1689936)

(d) ACAM-Medmamba (e) ACAM-ResNet (f) ACAM-ShuffleNet

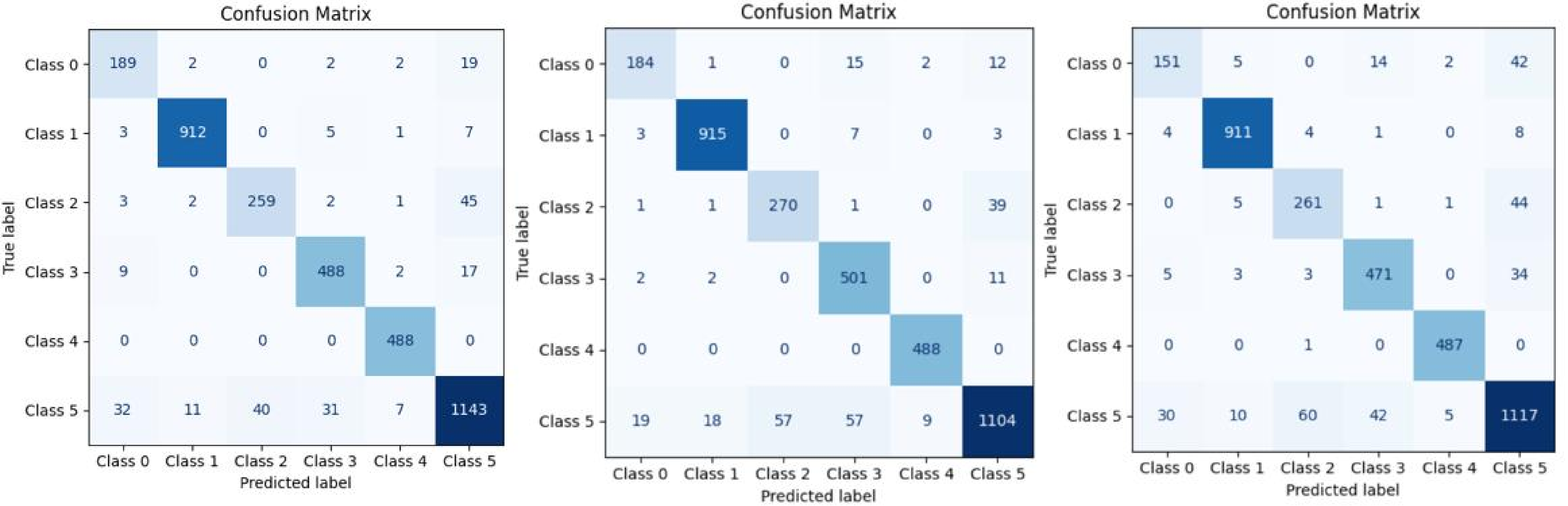

Supplement: Supplementary file 1 [file DataSheet1.zip › ACAM-main/confus2.pdf]
