# Supplementary figures and images for: Clinically guided adaptive contrast adjustment for fetal plane classification: a modular plug-and-play solution
Source: Front Physiol. 2025 Nov 13;16:1689936. doi: 10.3389/fphys.2025.1689936 (PMC12657189; doi:10.3389/fphys.2025.1689936)

(a) Medmamba  
ROC Curve

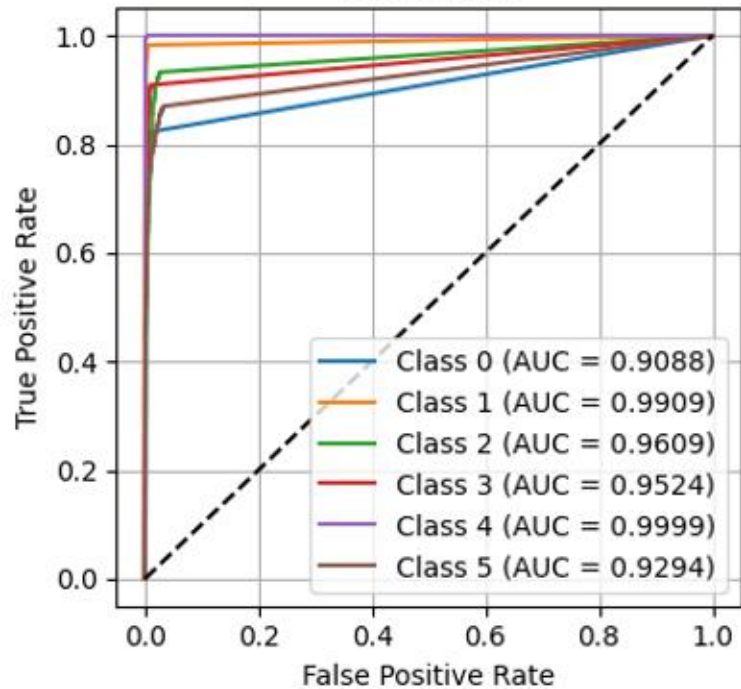

(b) ResNet  
ROC Curve

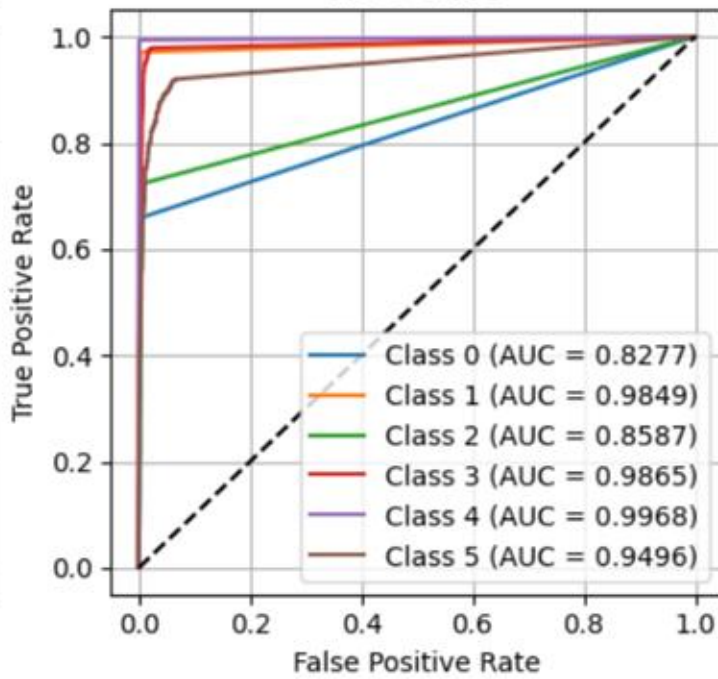

(c) ShuffleNet  
ROC Curve

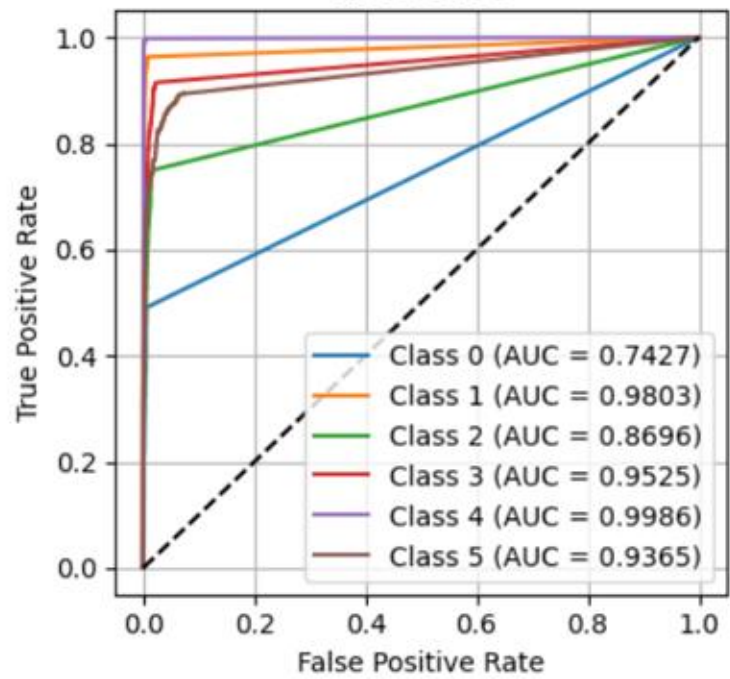

Supplement: Supplementary file 1 [file DataSheet1.zip › ACAM-main/ROC1.pdf]

(d) ACAM-Medmamba  
ROC Curve

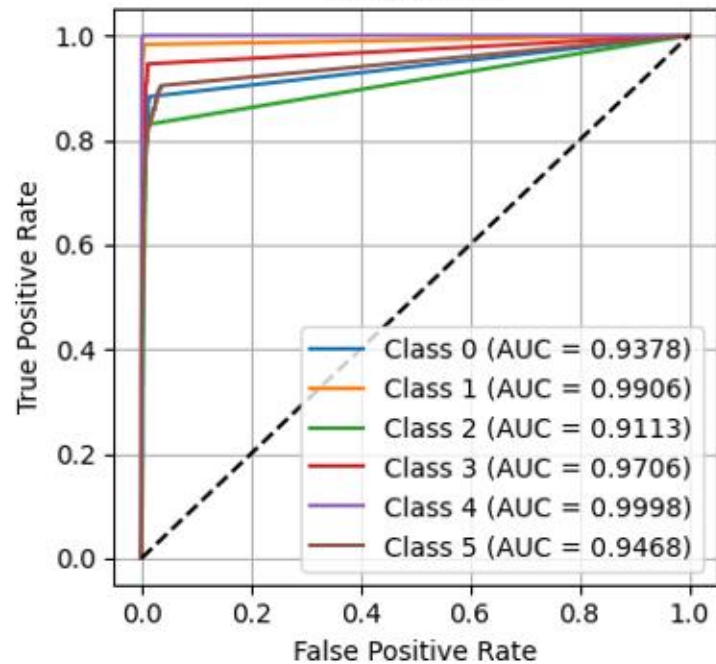

(e) ACAM-ResNet  
ROC Curve

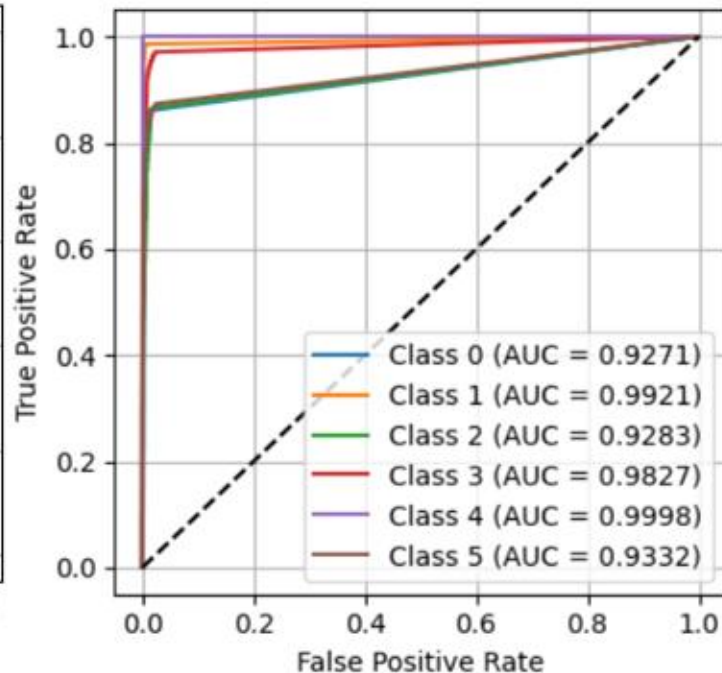

(f) ACAM-ShuffleNet  
ROC Curve

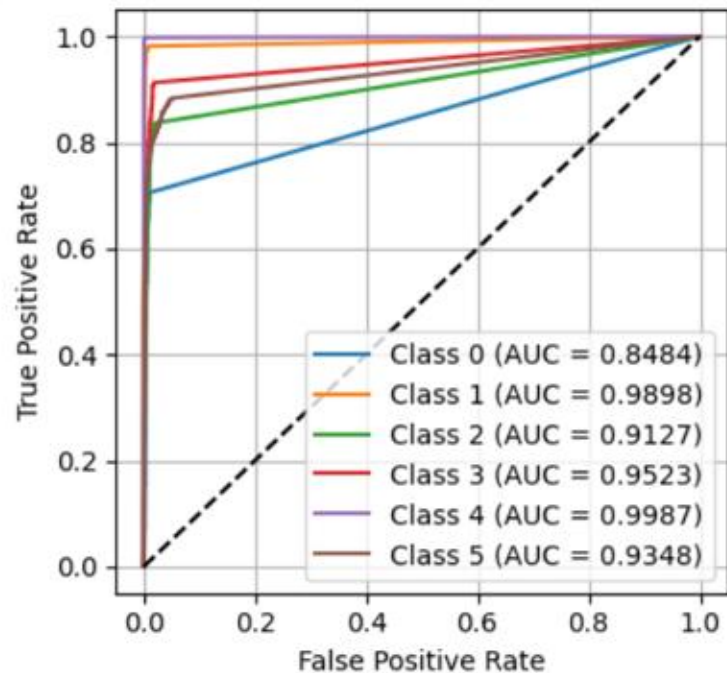

Supplement: Supplementary file 1 [file DataSheet1.zip › ACAM-main/ROC2.pdf]

(a) Medmamba  
Confusion Matrix

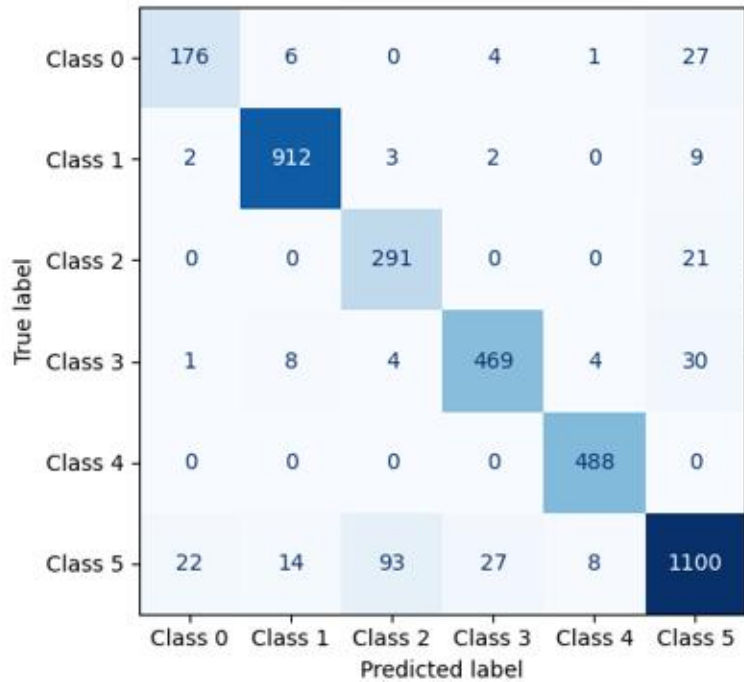

(b) ResNet  
Confusion Matrix

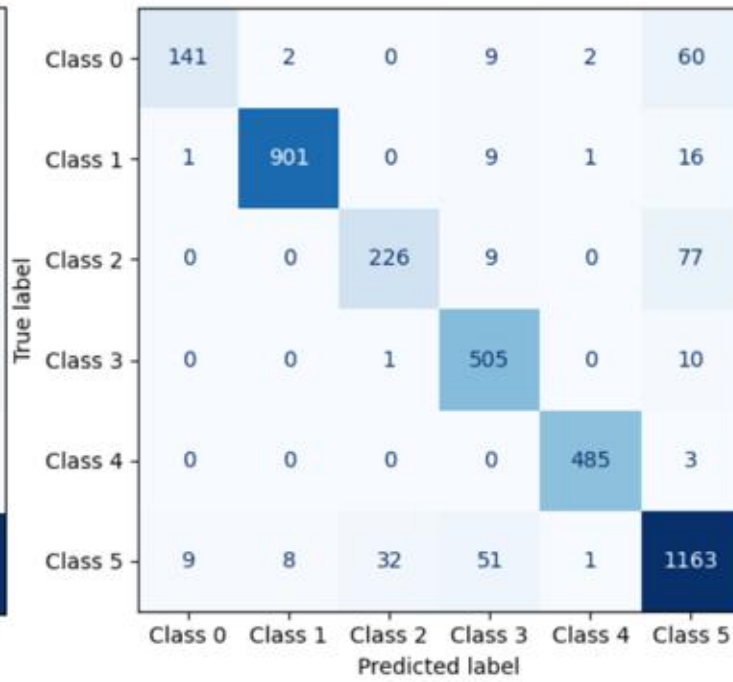

(c) ShuffleNet  
Confusion Matrix

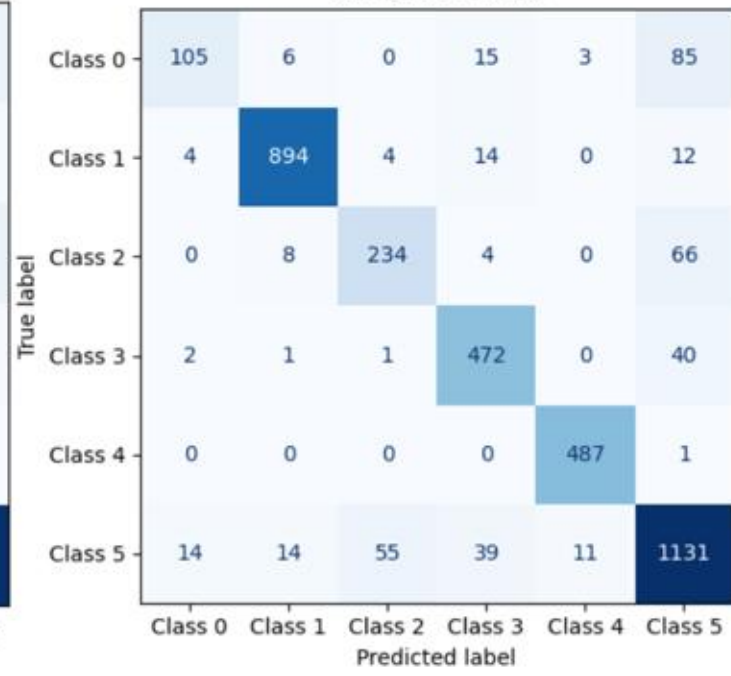

Supplement: Supplementary file 1 [file DataSheet1.zip › ACAM-main/confus1.pdf]

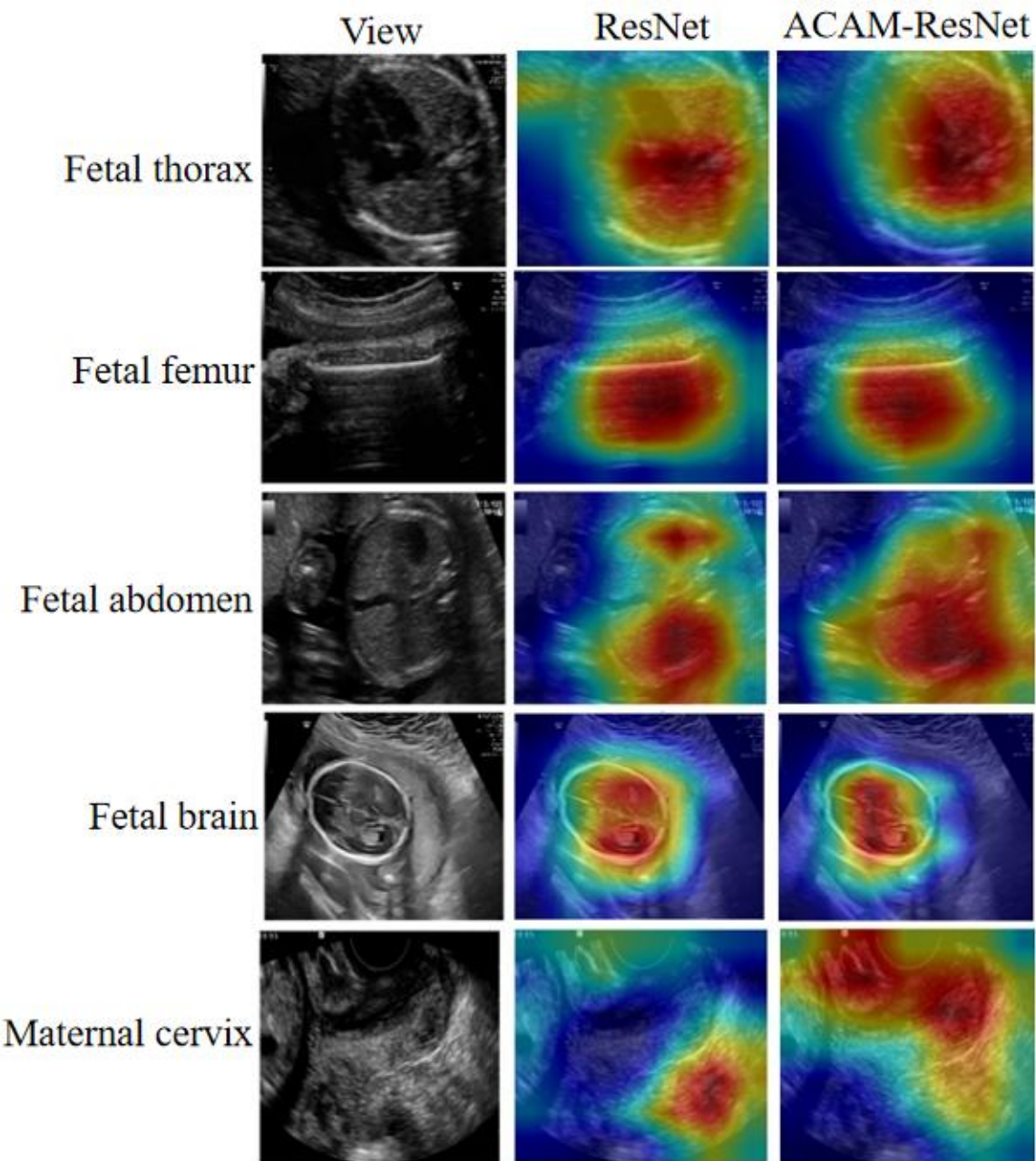

Supplement: Supplementary file 1 [file DataSheet1.zip › ACAM-main/heatmap1.pdf]

## Contrast Generation

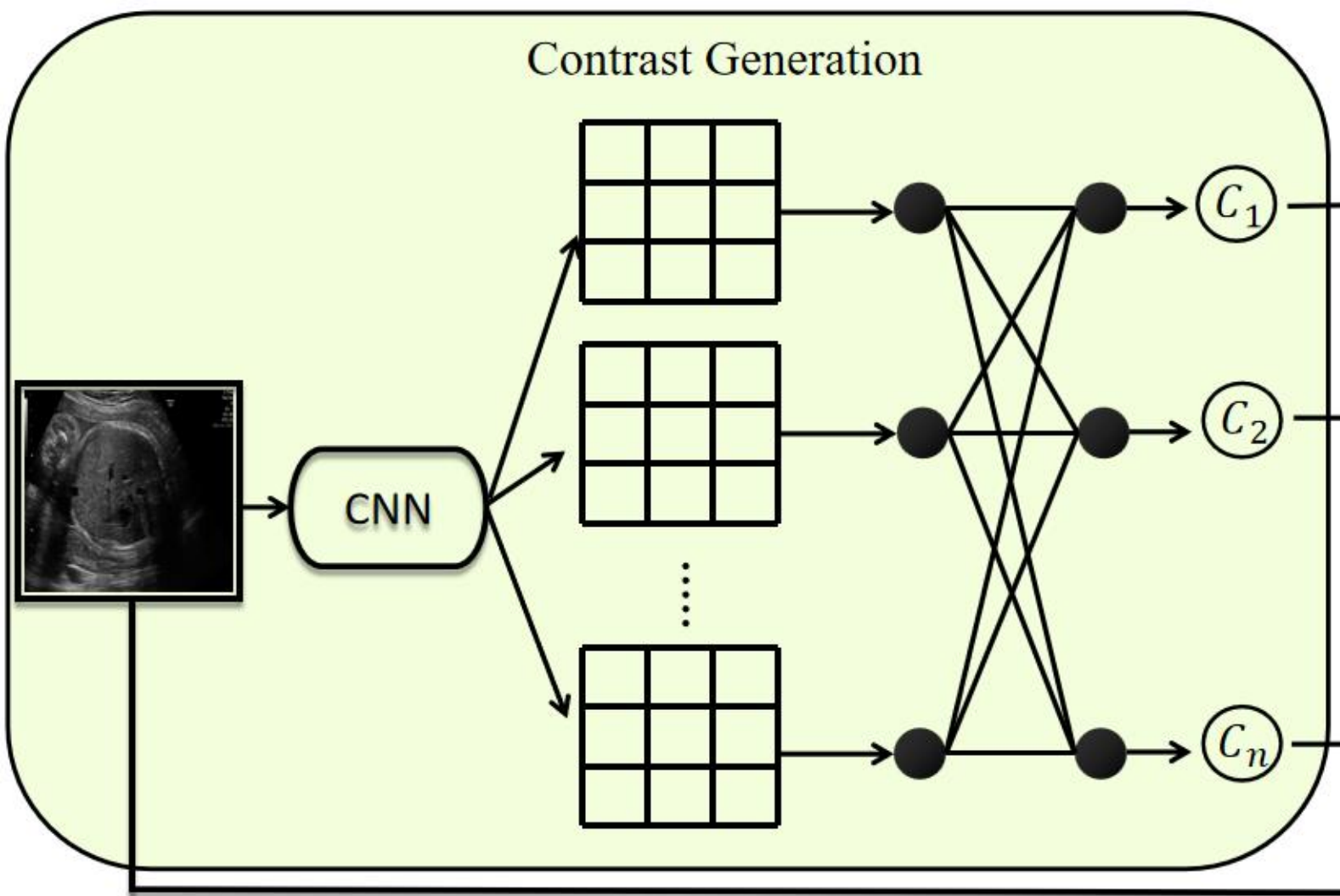

## Contrast Adjustment

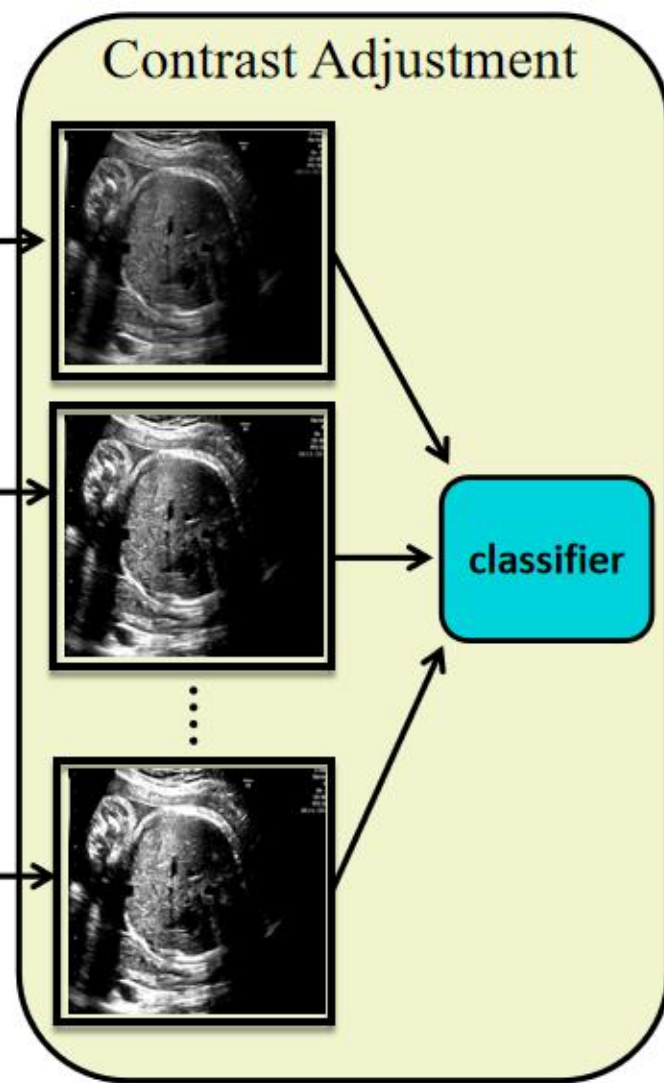

Supplement: Supplementary file 1 [file DataSheet1.zip › ACAM-main/module1.pdf]
